# Editorial: Epigenetic regulation of T cell function in type 1 diabetes

**DOI:** 10.3389/fimmu.2025.1773432

**Published:** 2026-01-09

**Authors:** Ubaid Ullah Kalim, Giuseppina Emanuela Grieco

**Affiliations:** 1 Turku Bioscience Centre, University of Turku and Åbo Akademi University, Turku, Finland; 2 Department of Medicine, Surgery and Neuroscience, University of Siena, Siena, Italy

**Keywords:** epigenetics, lncRNA - long noncoding RNA, microRNA, T cells, type 1 diabetes

Type 1 diabetes (T1D) is driven by a breakdown of immune tolerance that enables autoreactive T cells to destroy insulin-producing β cells. Although genetic susceptibility is well established, it is increasingly evident that epigenetic mechanisms—DNA methylation, histone modifications, chromatin accessibility, and non-coding RNAs—shape T cell identity, plasticity, and function in ways that critically influence disease initiation and progression. This Research Topic, *Epigenetic Regulation of T Cell Function in Type 1 Diabetes*, brings together four articles that collectively illustrate how epigenetic programs regulate diverse T cell populations and how their disruption contributes to autoimmunity. Together, these contributions provide a multidimensional view of how transcriptional and epigenetic landscapes integrate environmental cues, microbiome-derived signals, and inflammatory pathways to modulate T cell behavior in T1D.

The original research article by Jadon et al., PRMT5 regulates epigenetic changes in suppressive Th1-like iTregs in response to IL-12 treatment, examines how epigenetic regulators shape the plasticity and suppressive capacity of induced regulatory T cells (iTregs) exposed to a Th1-skewing cytokine environment. The authors demonstrate that IL-12 drives the differentiation of highly suppressive Th1-like iTregs and that this enhanced regulatory phenotype depends on the type II methyltransferase PRMT5. Using ChIP-seq, they identify PRMT5-mediated symmetric dimethylation at the Sirt1 promoter as a central mechanism repressing Sirt1 expression, thereby stabilizing Foxp3 and promoting durable regulatory function. Functionally, adoptive transfer of these Th1-like iTregs significantly improves survival in a mouse model of aplastic anemia—an autoimmune disorder characterized by T cell–mediated destruction of hematopoietic stem and progenitor cells—providing compelling evidence that targeted epigenetic remodeling can augment Treg-based immunotherapies. Although the disease model lies outside the context of T1D, the study highlights principles highly relevant to autoimmune diabetes: epigenetic enzymes act as lineage-stabilizing factors, and modulating their activity may reinforce regulatory networks capable of counterbalancing pathogenic T cell responses.

Two review articles in this Topic focus specifically on the role of epigenetics in shaping pathogenic or dysregulated T cell states in T1D. Jabri et al., *Linking epigenetic mechanisms of T cell dysfunction with pathophysiology of type 1 diabetes mellitus*, provide a comprehensive overview of how epigenetic modifications influence both CD4^+^ and CD8^+^ T cell subsets implicated in T1D. They summarize how FOXP3 promoter and TSDR hypermethylation destabilize Tregs, how activating histone marks at IFNG and IL17A enhancers fuel pro-inflammatory Th1 and Th17 responses, and how chromatin remodeling at cytotoxic effector loci primes autoreactive CD8^+^ T cells for β-cell destruction. The authors give special attention to the interplay between environmental factors—viral infection, dietary components, and microbiome-derived metabolites—and epigenetic dysregulation, highlighting how environmental exposures can accelerate immune activation in individuals carrying genetic risk alleles. Importantly, this review emphasizes that many T1D-associated variants map to noncoding regulatory elements, suggesting that altered epigenetic responsiveness at enhancers or promoters may underlie individual differences in disease trajectory. By integrating mechanistic and translational insights, this contribution underscores the promise of targeting epigenetic pathways for precision immunotherapy in T1D.

Complementing this perspective, Prasad et al., *The role of microRNAs and long non-coding RNAs in epigenetic regulation of T cells: implications for autoimmunity*, synthesize evidence on non-coding RNAs as important regulators of T cell epigenetic states. Their systematic review highlights consistent patterns across autoimmune diseases, including T1D: overexpression of miR-21, miR-148a, and miR-155; reduced expression of miR-146a and lncRNAs such as GAS5; and regulation of DNA methyltransferases, chromatin modifiers, and transcriptional repressors through miRNA–lncRNA networks. The authors emphasize that non-coding RNAs act at multiple levels—controlling chromatin accessibility, histone modification, and the stability of gene regulatory circuits—and that their dysregulation can drive T cells toward pathogenic phenotypes. Given that more than 90% of T1D-associated genetic variants lie in non-coding regions, this review highlights non-coding RNAs as both interpreters of genetic risk and potential therapeutic targets. Their call for single-cell multi-omics to capture ncRNA-chromatin interactions is timely and likely to accelerate the development of RNA-based therapeutics capable of restoring immune tolerance.

Finally, Fraser and Stadnyk, in *The emerging relationship between mucosal-associated invariant T cell populations and the onset and progression of type 1 diabetes*, expand the scope of epigenetic regulation by focusing on MAIT cells—innate-like T cells shaped heavily by microbial metabolites and developmental programming. Their review synthesizes evidence that gut dysbiosis, altered riboflavin-producing microbial communities, and impaired mucosal integrity may alter MAIT cell maturation and function. Importantly, MAIT cells in T1D show reduced circulating frequencies during recent onset, accumulation in inflamed pancreatic tissue, and transcriptional profiles consistent with exhaustion or altered effector potential. Although direct epigenetic studies of MAIT cells in T1D remain limited, the authors highlight strong links between microbial metabolites, MAIT cell development, and epigenetic imprinting during early life.

Across these four articles, several unifying concepts emerge. First, epigenetic regulation is not merely a downstream consequence of inflammation but a primary determinant of T cell identity, plasticity, and pathogenicity. Second, environmental signals—including cytokines, microbial metabolites, and metabolic stress—act through epigenetic pathways to modulate T cell responses in ways that may promote or prevent autoimmunity. Third, the integration of multi-omic approaches is beginning to reveal how risk variants, non-coding regulatory elements, and chromatin landscapes intersect to shape T cell dysfunction in T1D. Finally, these insights highlight new therapeutic opportunities: restoring Treg stability, modulating chromatin accessibility at inflammatory loci, or targeting non-coding RNAs may offer novel strategies to re-establish immune tolerance.

Together, the contributions in this Research Topic enrich our understanding of how epigenetic mechanisms govern T cell function in T1D and provide a foundation for future translational advances. We hope this Research Topic stimulates continued interdisciplinary research aimed at decoding the epigenetic architecture of autoimmune diabetes and developing targeted interventions to prevent or halt disease progression.

